# The Clinical Thermometer

**Published:** 1871-12

**Authors:** 


*The Clinical Thermometer.* By Z. C. McElroy, M. D.

The author speaks of the value of the instrument in the following manner:

"That the clinical thermometer, in connection with previous history and present rational symptoms, in any given case whatever, inasmuch as it reveals the actual condition of motion—that is chemical changes in the material of molecular forms of structure, or design, or material, ascending to or descending from forms of structure—the only thing pertaining to a human body, well or sick, alive or dead, which any remedial measure or agent whatever can influence—is, in the hands of the actual working practitioner, who can for the time being forget all mental conceptions connected with "disease," as popularly and professionally understood, an instrument of as much precision and certainty as any employed by civil engineers or mechanics in our own times."

